# Reference Values for Abdominal Circumference in Premature Infants

**DOI:** 10.3389/fped.2020.00037

**Published:** 2020-02-13

**Authors:** Héléna Setruk, Erika Nogué, Aurélie Desenfants, Olivier Prodhomme, Anne Filleron, Nicolas Nagot, Gilles Cambonie

**Affiliations:** ^1^Department of Neonatal Medicine, Arnaud de Villeneuve Hospital, Montpellier University Hospital Center, Montpellier, France; ^2^Department of Medical Information, Montpellier University Hospital Center, Montpellier, France; ^3^Department of Pediatrics, Carémeau Hospital, Nîmes University Hospital Center, Nîmes, France; ^4^Department of Pediatric Radiology, Arnaud de Villeneuve Hospital, Montpellier University Hospital Center, Montpellier, France

**Keywords:** abdominal circumference, abdominal distention, feeding intolerance, premature neonate, necrotizing enterocolitis

## Abstract

**Objectives:** Abdominal distention is a common indicator of feeding intolerance in premature newborns. In the absence of a precise definition, abdominal distention and its degree are highly subjective. The aim of this study was to construct references and smoothed percentiles for abdominal circumference (AC) and AC to head circumference (HC) ratio (AC/HC) in infants born between 24 weeks and 34 weeks of gestational age.

**Methods:** ACs and HCs were collected weekly in eutrophic premature infants without congenital abdominal or cerebral malformation. AC and HC charts were modeled using the LMS method, excluding measures associated with abdominal distention at clinical examination or intracranial abnormality at cerebral ultrasounds. Changes in AC and AC/HC over time were studied by repeated-measures analysis using mixed-effects linear models.

**Results:** A total of 1,605 measurements were made in 373 newborns with a mean gestational age of 31 [29–33] weeks and mean birth weight of 1,540 [1,160–1,968] g. Of these measurements, 1,220 were performed in normal conditions. Gestational age, postnatal age, singleton status, and respiratory support were significantly associated with AC and AC/HC. LMS curves were generated according to gestational age groups and postnatal age, with coherent profiles. AC/HC was 0.91 [0.86–0.95] in absence of abdominal distention. It was higher in cases of abdominal distention (0.95 [0.89–1.00], *p* < 0.001) and necrotizing enterocolitis (0.98 [0.93–1.07], *p* < 0.001).

**Conclusions:** References constructed for AC and AC/HC might be used to assess feeding tolerance in premature infants. AC/HC was more relevant than AC to rationalize the diagnosis of abdominal distention.

## Introduction

Achieving full enteral feeding of premature infants is a major and daily challenge in neonatal intensive care units (NICUs) (1). Early complete enteral feeding potentially decreases central line complications and length of hospital stay (2). In clinical practice, decisions regarding when to stop, maintain or increase enteral feeding are based on each infant's feeding tolerance, evaluated daily by the physicians in accordance with the nursing staff and the unit practices (3).

Feeding intolerance (FI) is a multifactorial phenomenon that occurs frequently during the hospital stay of a premature infant (4, 5). While FI is a benign condition in most cases, it may also be an initial manifestation of necrotizing enterocolitis (NEC) (6). The color and volume of gastric residuals, along with abdominal distention, are classic indicators of FI (7, 8). Yet residuals up to 3 mL may be physiological in these patients and, therefore, are poor predictors of FI and NEC (9, 10). An increase in abdominal circumference (AC) has been proposed as a surrogate of abdominal distention. A recent study compared AC to gastric residuals to measure FI and suggested that AC use may be associated with a shorter time to full enteral feeding (11). AC reference values, however, are not available in very premature infants (12). Furthermore, the relationship between an increase in AC and the visual appearance of abdominal distention has never been studied.

In our department, AC and head circumference (HC) are measured weekly in premature infants to evaluate feeding tolerance and monitor extrauterine growth. The first aim of the present study was to establish AC and AC/HC reference values in infants born between 24 and 34 weeks of gestational age. The second aim was to report AC and AC/HC values in cases of abdominal distention, NEC suspicion and confirmed NEC.

## Methods

### Patients

All preterm infants with a gestational age <35 weeks and hospitalized in the Department of Neonatal Medicine of Arnaud de Villeneuve Hospital, Montpellier University Hospital Center, between February 2014 and January 2016, were considered for this study.

In order to obtain sex-specific charts for AC and AC/HC in eutrophic premature infants, we excluded those with: (i) a birth weight <3rd percentile or >97th percentile on the Olsen curves (13), (ii) congenital abdominal (e.g., intestinal atresia or duplication, omphaloceles or gastroschisis, congenital diaphragmatic hernia, and cystic renal dysplasia) or cerebral (e.g., fetal ventriculomegaly) malformations, and (iii) a sex differentiation disorder.

### Intervention

Anthropometric measurements (weight, length, AC and HC) were performed weekly on a fixed day by the nurse caring for the patient.

**Figure 1:**
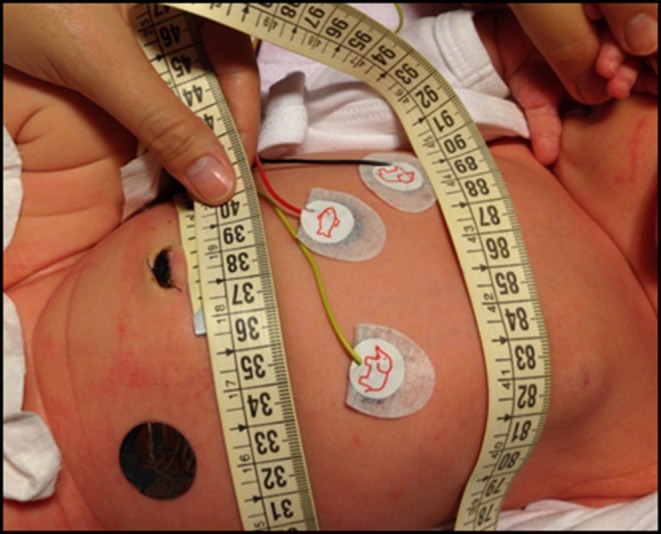
Illustration of AC measurement.

AC was measured by the bedside nurse with the infant in supine position, just before feeding, using a flexible plastic tape (Loutte, Saint-Quentin, France) positioned 1 cm above the umbilicus (14). The tape was not tight, allowing abdominal respiration. The measurement was recorded once, at the end of expiration, and was accurate to the millimeter value. This protocol for infant measurements had been implemented in the NICU before the beginning of the study. Several explanatory meetings were held and all nurses received a document with precise information and photographs on the measurements techniques (Figure 1). HC was measured at the largest fronto-occipital circumference with the same tape. Weight was obtained on a calibrated balance scale, included in the incubator for intubated patients (Caleo, Dräger, Lübeck, Germany) and outside the incubator for the non-intubated (Seca 213, Hamburg, Germany).

### Abdominal Examination and Transit Assessment

The abdominal examination was performed systematically following the weekly anthropometric measurements, and occasionally in the presence of signs compatible with NEC.

The nurse qualified the abdomen as normal or distended. Abdominal distention was defined by an unusual increase in abdominal volume, with or without one of the following signs: visibly dilated intestinal loops or a dilated venous/capillary network, palpable meconium stasis mass, abdominal wall discoloration or tenderness, or general discomfort of the neonate corresponding to a score >4 on the EDIN neonatal pain and discomfort scale (15).

According to our protocol, abdominal distention systematically required AC and HC measurements and a medical examination to confirm the distention and assess for general signs of NEC.

The abdominal assessment was used to classify the infants in one of the following groups: (i) normal abdomen (nurse assessment), (ii) absence of NEC suspicion (medical assessment), (iii) NEC suspicion requiring abdominal X-ray (medical assessment), and (iv) confirmed NEC (expert assessment).

Abdominal transit was considered adequate in the presence of at least one stool daily the week just preceding the assessment (16, 17).

### Actual Diagnosis

Two expert physicians who were not involved in the infants' management (OP and GC) analyzed the files of all patients suspected of NEC, 10 days after the initial digestive symptoms. On the basis of all the available evidence in the medical record, but blind from anthropometric data, they determined whether or not the digestive symptoms were related to NEC (18). This initial classification was subsequently compared to the patients' hospitalization records and no discrepancies were observed.

### Nutritional Policy in the NICU

Parenteral and enteral nutrition was prescribed daily in the NICU using an in-house computerized physician order entry system (19) that integrates the guidelines of the European Society for Pediatric Gastroenterology Hepatology and Nutrition (20). Enteral feeding with non-fortified human milk was started a few hours after birth at a volume of 10 ml/kg/day in cases of birth weight <1,000 g, small for gestational age, patent ductus arteriosus treated with cyclooxygenase inhibitor, catecholamine requirement, or severe respiratory distress syndrome, and it ranged up to 20 ml/kg/day in the absence of these conditions. Daily increases in enteral nutrition were 20 ml/kg in newborns weighing <1,800 g, and 30 ml/kg for the others provided their respiratory and cardiovascular status was stable. Fortifiers were added after enteral nutrition had reached a minimal volume of 70 ml/kg/day for all infants with a birth weight <1,500 g or born before 32 weeks of gestational age. Cessation of parenteral nutrition and withdrawal of the central venous catheter was performed as soon as well-tolerated enteral nutrition reached a volume of 90 to 120 ml/kg/day, according to birth weight.

Enteral nutrition was administered continuously by a nasogastric tube as long as the weight was ≤1,200 g. Post-pyloric enteral feeding was not used. Prefeed aspiration was systematically performed in infants fed discontinuously. In infants fed continuously, gastric residuals were assessed only in the presence of abdominal distention or signs compatible with NEC. Glycerin suppositories were occasionally administered if no transit had been observed for 48 to 72 h.

### Respiratory Support

Respiratory support at the time of the anthropometric measurements and abdominal examination was classified in five groups: (i) spontaneous ventilation (SV), (ii) high-flow nasal cannula (HFNC), (iii) nasal continuous positive airway pressure (nCPAP), (iv) conventional mechanical ventilation (CMV), and (v) high-frequency oscillatory ventilation (HFOV). The HFNC flow rate was set at 2 L/kg/min. The nCPAP level was set at 4–6 cmH_2_O.

### Statistical Analysis

Anthropometric measurements were excluded from the analysis in the following cases: (i) NEC; (ii) after abdominal surgery, whatever the cause; and (iii) any intracranial pathology increasing HC. In these cases, only data collected prior to the occurrence of these events were retained.

Smoothed percentile curves (3rd, 10th, 25th, 50th, 75th, 90th, and 97th percentiles) for AC and AC/HC were generated from the measures obtained in infants with normal abdominal assessment. These were obtained using the lambda-mu-sigma (LMS) method proposed by Cole and Green (21). The Rigby and Stasinopoulos algorithm determined the best model, and the goodness of fit of the final model was validated using the Q-statistics of Royston and Wright (22).

Changes in AC and AC/HC over time were also studied by repeated-measures analysis using mixed-effects linear models with an autoregressive lag 1 [AR(1)] covariate structure (23). The model took into account the random effect related to the mother due to multiple pregnancies. The interaction between gestational age and postnatal age was systematically assessed. Only significantly associated variables were entered into the model, i.e., *p* < 0.20 in univariate analysis and clinical relevance. A backward selection was implemented. Least square means (LSMeans) with their standard error (SE) and 95% confidence interval (95% CI) are reported.

The accuracy of detecting NEC based on AC/HC measurement was assessed using receiver operating curve (ROC) analysis. For this analysis, we compared the AC/HC values associated with normal examinations in patients who did not have NEC during hospitalization to the AC/HC values at the moment of diagnosis in 25 patients. AC/HC values associated with abdominal distention and those measured in patients after the occurrence of NEC were censored. The area under the ROC was calculated by the Hanley method and compared to the value 0.5 using Wilcoxon's W statistic. Statistical tests were performed 2-tailed and *p*-values < 0.05 were considered significant test results. Statistical analyses were conducted with SAS (version 9.4, SAS Institute, Cary, NC) and R software (R 2.3.4 for Windows).

## Results

### Population Characteristics

Six hundred and forty-seven preterm infants were admitted to the NICU during the study period.

**Figure 2:**
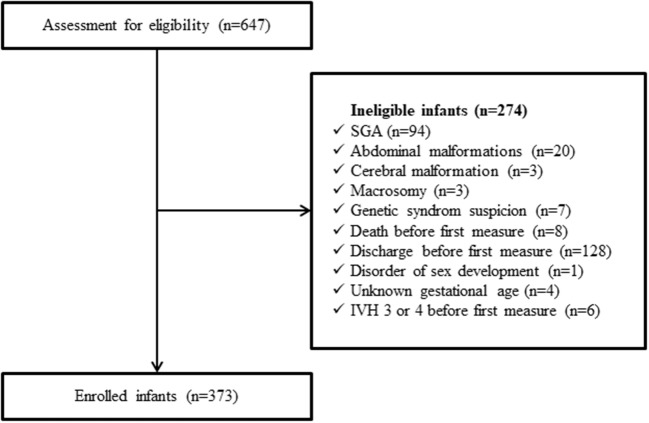
Flowchart of the population. SGA, small for gestational age; IVH, intraventricular hemorrhage.

Of these infants, 373 (57.6%) were eligible (Figure 2): 54% were born between 28 and 32 weeks, 14.5% before 28 weeks and 31.5% after 32 weeks. The median [IQR_25−75_] values for birth weight, length and HC were, respectively, 1540 [1,160–1,968] g, 41 [36–44] cm, and 28.5 [26–30.5] cm. The characteristics of the population are indicated in Table 1. Of the 373 infants, 224 (60%) remained in the department until home discharge, 134 were transferred to another care facility (36%), and death occurred in 15 cases (4%). The number of patients available for weekly anthropometric measurements until home discharge was 37 out of 54 for premature infants <28 weeks (69%), 126 out of 202 for premature infants 28–32 weeks (62%), and 61 out of 117 for premature infants >32 weeks (52%).

**Table 1:** Characteristics of infants with and without NEC suspicion according to the medical examination.

**Infants**	**Eligible population** ***n* = 373**	**Infants without** **NEC suspicion** ***n* = 278 (74.5%)**	**Infants with** **NEC suspicion** ***n* = 95** **(25.5%)**	**Infants with** **NEC** ***n* = 25** **(6.7%)**	***P*_**1**_**	***P*_**2**_**
**Birth data**						
Gestational age (weeks)	31 [29–33]	32 [30–33]	29 [26–31]	28 [26–29]	<0.001	<0.001
Birth weight (g)	1540 [1160–1,968]	1,705 [1,330–2,070]	1,145 [860–1,480]	980 [850–1,160]	<0.001	<0.001
Singleton *n* (%)	268 (72)	208 (75)	60 (63)	15 (60)	0.029	0.107
Male *n* (%)	199 (53)	150 (54)	49 (52)	14 (56)	0.688	0.844
**Postnatal**						
First stool (hours)	17 [7–40]	14 [6–35]	34 [11–50]	44 [16–61]	<0.001	<0.001
Inadequate transit FW^a^, *n* (%)	101 (29)	51 (19)	50 (56)	13 (54)	<0.001	<0.001
Parenteral nutrition (days)	6 [3–9]	5 [2–7]	11 [7–18]	18 [11–29]	<0.001	<0.001
Enteral feeding interruption, *n* (%)	67 (18)	19 (7)	48 (51)	22 (88)	<0.001	<0.001
Enteral feeding interruption (days)	0 [0–0]	0 [0–0]	1 [0–2]	3 [2–6]	<0.001	<0.001
Abdominal X-ray (number)	0 [0–1]	0 [0–0]	2 [1–4]	4 [3]	<0.001	<0.001

*Values are median [IQR_25−75_] or numbers (%). NEC, necrotizing enterocolitis; FW, first postnatal week*.

a *Missing data for 21 patients. P_1_, infants with vs. without NEC suspicion; P_2_, infants with NEC vs. infants without NEC suspicion*.

### Anthropometric Measurements

In all, 1,605 anthropometric measurements were analyzed, corresponding to 4.0 (2–6) measurements per infant. According to the gestational age classes, this number was 8 (6–10) for premature infants born before 28 weeks, 5 (3–6) for those born between 28 and 32 weeks, and 2 (1–3) for the those born after 32 weeks. The respiratory status at the moment of measurement was spontaneous ventilation in 52% of the cases, non-invasive support with HFNC or nCPAP in 45%, and invasive ventilation with CMV or HFOV in 3%.

### AC and AC/HC Data in Cases of Normal Abdominal Assessment

A total of 1,220 (76%) abdominal examinations were considered normal. Therefore, the ACs and HCs measured in this condition were used to generate the normal AC and AC/HC values. The median [IQR_25−75_] values for AC and AC/HC were, respectively, 27.0 [24.5–29.0] cm and 0.91 [0.86–0.95]. The median [IQR_25−75_] time interval between two measurements for these data was 7 (7) days.

**Figure 3:**
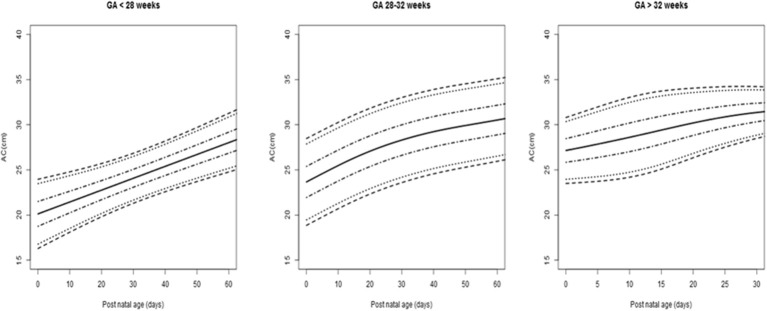
Abdominal circumference (AC) values (cm) for postnatal age (days) according to the different groups of gestational age (GA). Lines are 3rd, 10th, 25th, 50th, 75th, 90th, and 97th centiles.

**Figure 4:**
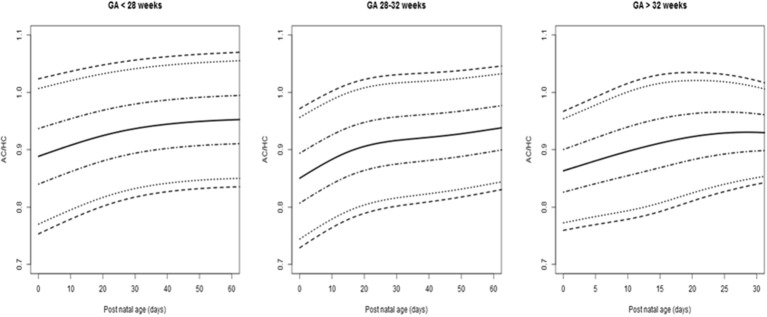
Abdominal circumference to head circumference ratios (AC/HC) for postnatal age (days) according to the different groups of gestational age (GA). Lines are 3rd, 10th, 25th, 50th, 75th, 90th, and 97th centiles.

**Figure 5:**
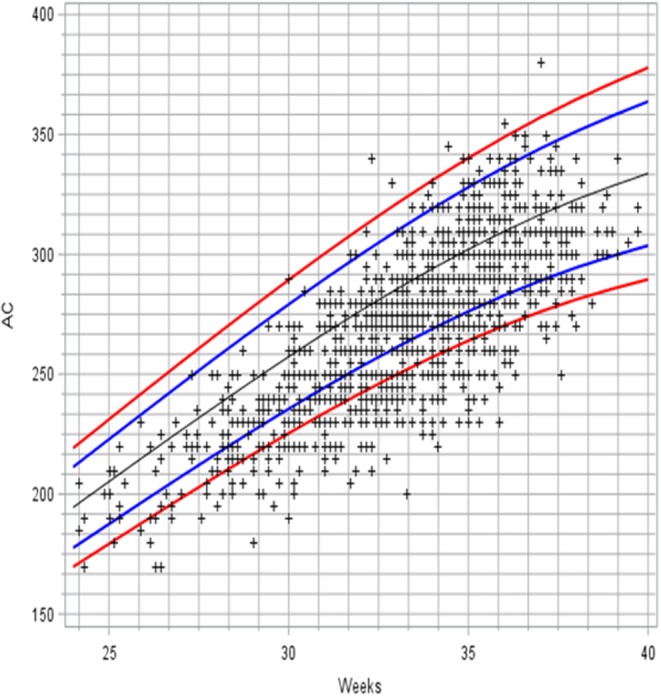
Projection of AC values associated with normal examinations on the French reference curve for fetal AC. Lines are 3rd, 10th, 50th, 90th, and 97th centiles.

The AC and AC/HC LMS curves were generated from 256 measurements for neonates born before 28 weeks (21%), 744 measurements for those born between 28 and 32 weeks (61%), and 220 for those born after 32 weeks (18%). They are presented in Figures 3, 4, respectively. Figure 5 shows the projection of our values on the reference curve recommended in our country to assess fetal AC by ultrasound (24).

#### Factors Associated With AC Values

Given the strong correlations between gestational age and weight, length, and HC at birth (Spearman correlation coefficients with gestational age, respectively, equal to 0.9, 0.86, 0.89), the model considered only the fixed effects of gestational age and postnatal age.

Several factors were associated with the AC values in univariate analysis: gestational age at birth (*p* < 0.0001), postnatal age (*p* < 0.0001), parenteral nutrition duration (*p* < 0.0001), respiratory support (*p* < 0.0001), singleton status (*p* = 0.01), and adequate transit the first week (*p* = 0.012). The effect of sex was not significant (*p* = 0.31).

Last, only gestational age, singleton status and respiratory support appeared significantly associated with the AC changes over time (Table 2). An AC model that included only two groups of respiratory support, i.e., with or without CPAP, revealed comparable LSMeans (*p* = 0.13).

**Table 2:** Factors associated with abdominal circumference (AC) values.

	**LSmeans**	**SE**	**95% CI**	***p***
**Gestational age**				<0.0001
<28 weeks	24.54	0.33	[23.89–25.19]	
28–32 weeks	28.39	0.23	[27.95–28.83]	
>32 weeks	31.61	0.31	[31.01–32.21]	
**Postnatal age**				<0.0001
Week 1	23.53	0.21	[23.13–23.94]	
Week 2	24.34	0.22	[23.92–24.76]	
Week 3	25.61	0.23	[25.16–26.06]	
Week 4	26.71	0.24	[26.24–27.19]	
Week 5	27.39	0.25	[26.89–27.89]	
Week 6	28.27	0.27	[27.74–28.79]	
Week 7	29.03	0.29	[28.47–29.60]	
Week 8	30.30	0.32	[29.67–30.93]	
Week 9	30.75	0.33	[30.11–31.40]	
Week 10	31.56	0.39	[30.79–32.34]	
Week 11	32.49	0.45	[31.60–33.34]	
**Singleton/twins**				0.0042
Singleton	28.57	0.21	[28.16–28.97]	
Twins	27.80	0.29	[27.23–28.36]	
**Respiratory support**				<0.0001
SV	28.91	0.17	[28.57–29.25]	
HFNC/nCPAP	28.35	0.20	[27.96–28.75]	
CMV/HFOV	27.28	0.41	[26.48–28.09]	

*LSMeans, least square means; SE, standard error; 95% CI: 95% confidence interval; SV, spontaneous ventilation; HFNC, high-flow nasal cannula; nCPAP, nasal continuous positive airway pressure; CMV, conventional mechanical ventilation; HFOV, high-frequency oscillatory ventilation*.

#### Factors Associated With AC/HC Values

In univariate analysis, associations were found between the AC/HC ratios and gestational age at birth (*p* = 0.011), postnatal age (*p* < 0.0001), respiratory support (*p* = 0.0006), and singleton status (*p* = 0.003). These four variables were included in the final model (Table 3). Sex was also entered into the model but not retained.

**Table 3:** Factors associated with AC/HC, ratio of abdominal circumference (AC) to head circumference (HC).

	**LSmeans**	**SE**	**95% CI**	**p**
**Gestational age**				0.016
<28 weeks	0.92	0.008	[0.90–0.94]	
28–32 weeks	0.90	0.006	[0.89–0.91]	
>32 weeks	0.91	0.008	[0.90–0.93]	
**Postnatal age**				<0.001
Week 1	0.86	0.006	[0.85–0.87]	
Week 2	0.88	0.006	[0.87–0.89]	
Week 3	0.90	0.006	[0.89–0.91]	
Week 4	0.91	0.007	[0.90–0.92]	
Week 5	0.91	0.007	[0.90–0.93]	
Week 6	0.92	0.008	[0.90–0.93]	
Week 7	0.92	0.008	[0.90–0.94]	
Week 8	0.93	0.009	[0.91–0.95]	
Week 9	0.93	0.010	[0.91–0.95]	
Week 10	0.93	0.012	[0.91–0.95]	
Week 11	0.93	0.014	[0.90–0.95]	
**Singleton/twins**				0.006
Singleton	0.92	0.006	[0.91–0.93]	
Twins	0.90	0.007	[0.89–0.92]	
**Respiratory support**				0.001
SV	0.92	0.004	[0.91–0.93]	
HFNC/nCPAP	0.93	0.005	[0.92–0.94]	
CMV/HFOV	0.88	0.012	[0.86–0.91]	

*LSMeans, least square means; SE, standard error; 95% CI, 95% confidence interval; SV, spontaneous ventilation; HFNC, high-flow nasal cannula; nCPAP, nasal continuous positive airway pressure; CMV, conventional mechanical ventilation; HFOV, high-frequency oscillatory ventilation*.

### Abnormal Abdomen Assessments

**Figure 6:**
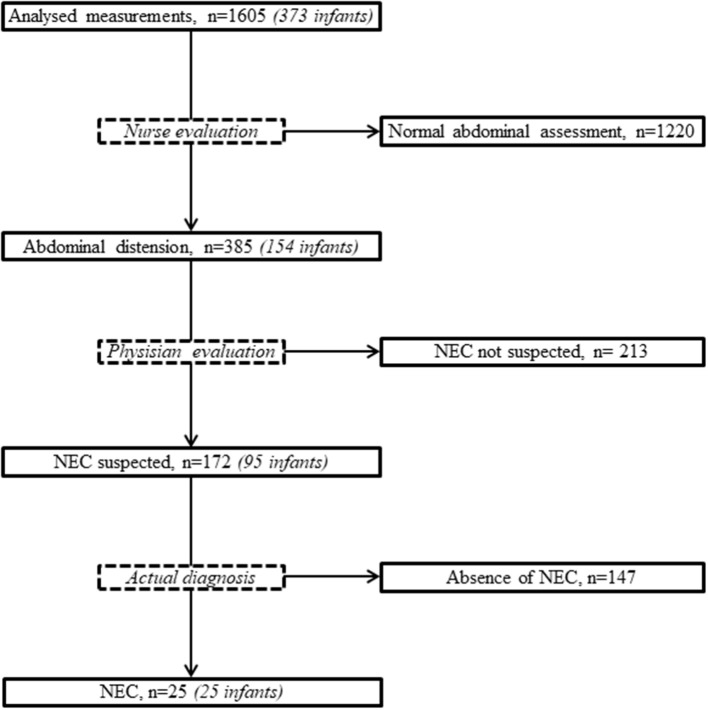
Flowchart of abdominal assessments. NEC, necrotizing enterocolitis.

At the nurses' evaluations, the abdomen was considered distended in 385 cases, which corresponded to 154 infants. Among them, the physicians suspected NEC in 172 cases, which corresponded to 95 infants, i.e., 25.5% of the population analyzed (Figure 6).

### NEC Suspicion

The infants with at least one episode of NEC suspicion were born more prematurely and required longer parenteral nutrition. Their abdominal transit was less regular in the first postnatal week, and enteral feeding was more frequently interrupted (Table 1). The median number of abdominal X-rays for NEC suspicion during the hospital stay was higher in the most preterm infants: 2 (1–4) in infants born before 28 weeks, 0 [0–1] at 28–32 weeks, and 0 [0–0] after 32 weeks (*p* < 0.001).

The examinations performed in the presence of abdominal distention found abnormal local and general signs more frequently and indicated higher AC/HC. These results were even more marked in the presence of NEC suspicion (Table 4).

**Table 4:** Characteristics of the examinations according to the nursing and medical examinations.

**Examinations** **(*n* = 1,605)**	**Normal assessment** **(*n* = 1,220)**	**Presence of abdominal distention**		**Confirmed NEC** **(*n* = 25)**	***P_**1**_***	***P_**2**_***
		**NEC not suspected** **(*n* = 213)**	**NEC** **suspected** **(*n* = 172)**			
**Digestive signs and symptoms** ***n*** **(%)**						
Inadequate transit, PW^a^	169 (14)	50 (23)	62 (36)	8 (32)	<0.001	0.018
Bloody stools	1 (0.1)	0 (0.0)	31 (18)	5 (20)	<0.001	<0.001
Bilious aspirates	17 (1.4)	4 (1.9)	12 (7.0)	2 (8.0)	<0.001	0.037
Vegetative signs^b^	24 (2.0)	3 (1.4)	11 (6.4)	2 (8.0)	0.004	0.072
Discomfort	6 (0.5)	1 (0.5)	11 (6.4)	10 (40)	<0.001	<0.001
**Anthropometric measurements**						
AC (cm)	27.0 [24.5–29.0]	25.5 [24.0–28.0]	25 [23.0–28.0]	26 [25–28]	<0.001	0.151
AC/HC	0.91 [0.86–0.95]	0.94 [0.89–0.98]	0.96 [0.89–1.01]	0.98 [0.93–1.07]	<0.001	<0.001

*Values are median [IQR_25−75_] or numbers (%). NEC, necrotizing enterocolitis; PW, previous week; AC: abdominal circumference; HC, head circumference*.

a *Missing data for 76 examinations*.

b *Apnea, bradycardia, thermal instability. P_1_, difference between the first three groups; P_2_, confirmed NEC vs. normal assessment*.

#### Confirmed NEC

After 10 days of evolution, the two experts diagnosed NEC in 25 neonates, corresponding to 6.7% of the patients and 26.3% of the patients with suspected NEC. At initial examination, local and general signs were not different from those observed in the NEC suspected group (Table 4).

**Figure 7:**
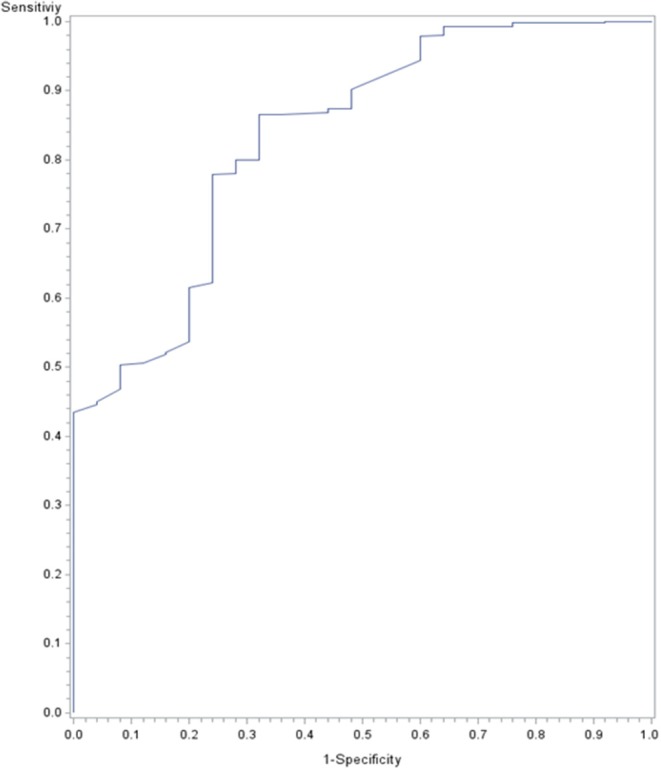
Receiver operating curves for the abdominal circumference (AC) to head circumference (HC) ratio (AC/HC) for detecting necrotizing enterocolitis (NEC). AC/HC values associated with normal examinations in patients who did not have NEC during hospitalization were compared to AC/HC values at the moment of diagnosis in patients with NEC.

The predictive value of AC/HC was tested in the normal assessment and NEC groups (Figure 7). The area under the ROC curve for AC/HC to detect NEC was 0.83 (95% CI 0.73–0.93, *p* < .000001). A cut-off value of 0.98 had a sensitivity of 68%, a specificity of 86%, a positive likelihood ratio of 4.82 and a negative likelihood ratio of 0.37. Infants with the most severe forms of NEC—that is, those with Bell's stage 3 or death as a consequence of NEC—had values of AC/HC that were comparable to those observed in the other patients with NEC (data not shown).

## Discussion

This study proposes for the first time reference values for AC and AC/HC in very preterm infants. In order to adapt to the situations encountered in NICUs, the data take into account the degree of prematurity and postnatal age. The references proposed here are of clinical interest to confirm the impression of abdominal distention, prompt the clinician to conduct further examinations to rule out or diagnose NEC, and evaluate the somatic growth of premature newborns.

Few studies have reported AC reference data for preterm infants. Rodriguez et al. established charts for AC in term and near-term Caucasian newborns. However, only singleton newborns at a gestational age >35 weeks were considered in this investigation (12). Meldere et al. measured AC in 220 infants born before 35 weeks of gestation, but this study included a single measurement within 30 min of birth (25). More recently, AC was assessed among other length and circumferential measures in preterm neonates, but the resultant models were only reliable from 33 weeks post-menstrual age (26). In this study, we prioritized the inclusion of very-preterm infants, because it is mainly these patients who are at the highest risk of FI and NEC (17). The distribution of birth terms in our sample was quite comparable to that observed in the cohort of premature infants born in our country in 2011, with about 70% of newborns in both studies born before 32 weeks (27). Compared to AC growth during intrauterine life, our data suggested a downward shift in values, with 18% of the points below the 3rd percentile. This result is consistent with previous observations showing that a majority of preterm infants fail to approximate *in utero* growth rates during their stay in neonatology (28, 29).

As observed in two previous studies, the influence of sex on AC values was nil or very weak in the preterm neonates, and thus the AC curves can be applied to these patients regardless of sex (12, 26). Mihatsch et al. found a linear relationship between AC and body weight in 42 preterm infants on full enteral nutrition (14). As our study shows, however, factors other than weight influenced the value of AC in these patients, notably the gestational age group and the postnatal age. The linear increase in AC the first 2 months in the group born before 28 weeks was only observed in the first month in the group born between 28 and 32 weeks. This observation was consistent with studies suggesting greater total and visceral fat accumulation from birth to hospital discharge in the most premature newborns compared with preterm infants of higher gestational ages (30). Clinicians frequently assume that nCPAP generates abdominal distention in the premature newborns. In their metanalysis, Lemyre et al. reported 10 to 15% of abdominal distention requiring feed cessation in preterm neonates supported with nasal intermittent positive pressure ventilation (nIPPV) or nCPAP after extubation (31). Heimann et al. proposed the ratio of AC to body weight as an objective parameter of abdominal distention and found that nCPAP had no significant effect on this ratio during the first postnatal month (32). Similarly, we observed no AC increase with nCPAP using LSMeans, whether nCPAP was individualized or associated with HFNC in a non-invasive ventilation group.

Abdominal or waist circumferences have previously been studied in relation to the infant's size (30) or weight (14). Several factors prompted us to express AC in relation to HC. HC is easier to measure then length, and its postnatal growth during hospitalization has been associated with neurodevelopmental outcome (33). In addition, it seemed more consistent to compare circumferences to translate the clinical impression of an increase in the abdominal volume. Higher AC/HC was observed at birth in infants with the lowest gestational ages, and the postnatal increase in this ratio plateaued at the end of the first month in the three groups. These data confirm the visual impression of a more pronounced abdominal distention in the most premature newborns, independently of any gastrointestinal symptoms.

According to a recent study from the UK National Neonatal Research Database, abdominal distention was the most frequent finding among infants with NEC (34). In our patients, we observed a combination of symptoms in addition to increased AC/HC. The predictive value of AC/HC was quite modest and this ratio alone cannot be used to discriminate between infants with and without NEC. Quantifying distention using AC/HC may nevertheless be helpful by increasing the likelihood of diagnosis. Regardless of the degree of prematurity, 0.98 could be given to nurses as a threshold value, above which they should seek medical examination of the infant, especially if other digestive or general signs compatible with this diagnosis are present.

In a recent prospective nationwide population-based study, irregular intestinal transit in the first week of life emerged as an individual risk factor for NEC (17). Our study, however, did not confirm that this rather common symptom clearly discriminated between infants with NEC suspicion and infants with confirmed NEC. In addition, the efficacy of prokinetic medications (35) or glycerin laxatives (36) to prevent or treat early FI has not been demonstrated in very low birth weight infants.

## Limitations

The limitations of this study include the single-center design and the limited sample size.

In order to obtain standardized curves for AC and AC/HC, we excluded neonates with low weight for gestational age. The exclusion of this population means that our references cannot be applied to these infants at higher risk of gastrointestinal complications, including NEC (37, 38), and for whom few data are available to guide enteral feeding (39). Further investigations would be necessary to specify the AC values for infants born small for gestational age and those with abnormal antenatal umbilical artery Doppler waveforms.

We assumed that measurements of AC and HC would not be subject to daily variation for a majority of our population, and we thus opted for weekly measurements. This choice also took into consideration the objective of minimal destabilization for the patient, as well as the possibility of collecting these data as part of the usual care by the nursing staff. Daily measurements would potentially have allowed us to document more precisely the evolution of these anthropometric measurements in patients developing gastrointestinal symptoms and to propose more accurate predictive criteria for NEC, based on their short-term evolution.

We did not assess intra- and inter-observer reliabilities for AC and HC. West et al. found reliabilities between 80 and 99% for routine circumference measurements, with relative technical error of measurement values all below 4% (40). In a multi-center cohort study, Abdel-Rahman et al. (26) found that AC was one of the most reproducible circumferential measurements, with an inter-rater reliability of 0.99 [0.98–0.99]. Circumferences are routinely measured in the department and, before the study began, we had provided numerous training sessions for nurses, with the distribution of explanatory material, in order to limit these errors.

## Conclusion

FI is frequent in preterm infants, generally related to the immaturity of gastrointestinal function. In the face of impending NEC, interpreting the signs of FI, notably the degree of abdominal distention, is highly subjective. This study provides information about normal AC values in these preterm patients according to gestational age and postnatal age. The expression of AC in relation to HC appears useful to rationalize the diagnosis of abdominal distention as a first step in targeting those patients who may require a more thorough abdominal examination and possibly additional explorations with X-ray and/or US. Future studies are required to assess whether these references can help reduce the delayed attainment of full enteral feeding and the prolonged intravenous nutrition supply in this population.

## Data Availability Statement

The raw data supporting the conclusions of this article will be made available by the authors, without undue reservation, to any qualified researcher.

## Ethics Statement

The studies involving human participants were reviewed and approved by Institutional review board of Montpellier University Hospital. Written informed consent to participate in this study was provided by the participants' legal guardian/next of kin.

## Author Contributions

HS, AD, AF, and GC conceptualized and designed the study, contributed to the search for published works, carried out the data acquisition and interpretation, and drafted and finalized the report. EN and NN performed the data analysis, contributed to the data interpretation, and critically revised the report. OP and GC analyzed the files of all patients with suspected NEC and, blind from the anthropometric data, classified the patients according to the Bell stage if required. All the authors have read and approved the final manuscript.

### Conflict of Interest

The authors declare that the research was conducted in the absence of any commercial or financial relationships that could be construed as a potential conflict of interest.

## Abbreviations

AC: abdominal circumference
FI: feeding intolerance
HC: head circumference
NEC: necrotizing enterocolitis
NICU: neonatal intensive care unit.
